# Intravenous paracetamol pharmacokinetics in neonates: a pooled analysis

**DOI:** 10.1186/cc9766

**Published:** 2011-03-11

**Authors:** K Allegaert, G Palmer, B Anderson

**Affiliations:** 1 University Hospitals Leuven, Belgium; 2 Royal Children's Hospital, Melbourne, Australia; 3 University of Auckland, New Zealand

## Introduction

The aim of this study was to describe paracetamol pharmacokinetics in neonates, to determine its covariates and suggest a dosing regimen for neonates (28 to 44 weeks postmenstrual age (PMA)).

## Methods

A population PK analysis of paracetamol time-concentration profiles (943 observations) from 158 neonates (27 to 45 weeks PMA) was undertaken using nonlinear mixed-effects models. Data from three earlier published studies involving neonates given either i.v. propacetamol or paracetamol were pooled with newly collected observations during repeated i.v. paracetamol administration (*n *= 60, 343 observations, PARANEO study) [1-3].

## Results

A two-compartment linear disposition model was used. Population parameter estimates (between-subject variability, %) were central volume (V1) 51.9 (21.6%) l/70 kg, peripheral volume of distribution (V2) 22.7 l/70 kg, clearance (CL) 5 (40%) l/hour/70 kg and inter-compartment clearance (Q) 16.2 l/hour/70 kg. About one-half (60.9%) of the overall CL variance is predictable from covariates. Weight was used to predict size and this was the major covariate (57.5%). Clearance expressed as mg/kg/hour increases only slightly with PMA (0.138 at 28 weeks, 0.167 l/hour/kg at 44 weeks PMA), contributing only 2.2% of variance within this cohort. Unconjugated bilirubin contributed only an additional 1.2% of variance.

## Conclusions

An increased volume of distribution supports the use of a loading dose when instigating paracetamol therapy in neonates while size is the major covariate of clearance. Clearance matured slowly in this cohort and a mean paracetamol serum concentration of 11 mg/l is achieved in neonates (28 to 44 weeks) given a standard dose of paracetamol of 10 mg/kg/6 hours. Based on these estimates, we suggest a loading dose of 20 mg/kg followed by 6-hourly dosing (10 mg/kg) within the age range evaluated.
